# Distractor avoidance and early quitting in visual search

**DOI:** 10.3758/s13414-025-03188-2

**Published:** 2025-12-02

**Authors:** Anjum Shaikh, Idah Mbithi, Maiko Okamura, Skylar Rice, Lily Rosan, Fabio Solorzano Quesada, Trafton Drew, Brennan Payne, Jeff Moher

**Affiliations:** 1 University of Connecticut School of Medicine, Farmington, CT USA; 2 Psychology Department, Connecticut College, 270 Mohegan Avenue, New London, CT 06320 USA; 3 Sirona Medical, San Francisco, CA USA; 4 Department of Psychology, University of Utah, Salt Lake City, UT USA

**Keywords:** Visual search, Eye movements and visual attention, Attentional capture

## Abstract

In the current study, we examined the mechanisms underpinning how salient distractors produce early quitting in visual search. Participants completed a simple visual search task and indicated whether a target was present or absent. When salient distractors were present, fewer eye movements occurred before target-absent responses, and less of the display area was searched. Surprisingly, participants actively avoided directing eye movements towards the distractor. Still, salient distractors increased both search errors, which were committed when the target was never fixated, and decision errors, which were committed when the target was fixated but not detected. Our results demonstrate that salient distractors trigger early quitting by reducing the amount of information that observers extract from the search image and disrupting search guidance.

## Introduction

Many adults in North America may recall completing *Where’s Waldo* puzzles (Handford, 1987) in children’s books when they were younger. In these books, the reader is tasked with finding Waldo, a gentleman dressed in a red-and-white striped shirt, winter hat, glasses, and jeans. Waldo is hidden in a scene containing dozens of people and objects. To add to the complexity of the puzzle, many of the other individuals or objects possess the same features as Waldo, notably the red-and-white striped clothing. Even though these puzzles are marketed to children, *Where’s Waldo* is, at its core, a challenging visual search task. In the real world, medical imaging, x-ray baggage screening, and other high-stakes tasks rely heavily upon the same principle as *Where’s Waldo*: visually sifting through irrelevant objects to find a possible target. The ability to conduct efficient and accurate visual search is critical to various occupations in which making mistakes could be costly.

One factor that can impact the visual search process is the presence of salient but irrelevant distractor objects. For example in Theeuwes’ (1992) influential study, participants searched for a target that was always a green circle. Other non-target items differed from the target (e.g., green squares amongst a green circle target). *Color singletons*, or uniquely colored non-targets, were sometimes present (e.g., a red square). These were considered *salient distractors*. The increased response times (RTs) to find a target observed in the presence of a salient distractor demonstrate the concept of *singleton-presence cost* – that is, task-irrelevant color singletons elicit *attentional capture*. In the decades since, there has been a long-standing debate regarding whether attentional capture by salient stimuli is automatic or subject to top-down control (see, e.g., Luck et al., 2021, Theeuwes, 2025, for reviews).

Attentional capture can have serious real-world consequences and cause distraction even in high-stakes scenarios. For example, in one recent study examining motor vehicle accidents during three periods when billboards were present, removed, and restored, researchers reported that billboard removal was associated with a 30‒40% decrease in crashes with injuries, whereas billboard restoration was associated with a 40‒50% increase in crashes with injuries (Gitelman et al., 2019). Salient distractors can also disrupt eye movements. Theeuwes et al. (1998) reported that non-target distractors appearing at a delayed onset could redirect planned eye movements to their location, resulting in oculomotor capture.

The literature on attentional capture almost exclusively employs the use of distractors when target prevalence is 100%, similar to how Waldo is always present somewhere in each of the visual search scenes in the children’s books. In these visual search studies, the participant is typically required to discriminate a feature of the target object to demonstrate that they have located it. However, this scenario is not always applicable to real-world scenarios. For example, in digital breast tomosynthesis (three-dimensional mammography), the modality captures one to three tumors (target) for every 1,000 cases read by radiologists (Østerås et al., 2019). When targets are not present on each trial, an additional decision component is present. That is, participants must determine how long they want to examine the visual search scene before reaching a quitting threshold and terminating the search by indicating that no target is present (e.g., Chun & Wolfe, 1996; Moran et al., 2013; Wolfe & Van Wert, 2010).

To better understand how distractors might impact a task involving quitting thresholds, Moher (2020) investigated salient distractors in a *target-detection search* where targets were not always present. In this study, participants searched for a vertical blue rectangle target among non-target blue rectangles tilted 30° to the right or left. The vertical target was present on a randomly selected 50% of all trials. On an independently randomly selected 50% of all trials, a salient distractor was presented that differed from other objects in color (red), size (four times as large), and onset (100 ms after the appearance of other objects). When targets were present, a salient distractor increased RTs similar to prior research and consistent with the idea of attentional capture (e.g., Theeuwes, 1992). However, when targets were absent, the presence of a salient distractor *reduced* RTs. Furthermore, salient distractors increased miss errors when targets were present.

Moher (2020) termed the target-absent distractor phenomenon the *distractor-induced quitting effect*. That is, when salient distractors appear, observers may be more likely to quit the visual search early, and, as a result, be more likely to fail to report a target. A number of other recent studies have found evidence for similar effects in which distractors can impact quitting thresholds in visual search (e.g., Lawrence & Pratt, 2022; Lawrence et al., 2023a, b; Lui et al., 2024; Wu & Pan, 2022). However, all of these studies have employed simple key-press responses, meaning that we still have limited information about how exactly salient distractors are impacting real-time search behavior.

In the present study, we used eye tracking to elucidate the mechanisms involved in distractor-induced quitting, using the same task as Moher (2020) in which participants are asked if a vertical blue line is present on each trial, with a large, delayed onset, tilted red line salient distractor present on a subset of trials. We focused primarily on five questions that we believe can add valuable knowledge and context to our understanding of distractor-induced quitting. Each question is listed below:

1. *Does distractor-induced quitting replicate in a lab-based setting?* Our previous research, and others who have studied this phenomenon, have relied largely on online data collection (e.g., Lawrence & Pratt, 2022; Lawrence et al., 2023a, b; Moher, 2020). In the present study, we are replicating the design of Moher (2020) measuring eye movements and key-presses. We predict that the effects will replicate in a laboratory-based study, similar to previous research showing that online studies and laboratory studies exhibit similar patterns (e.g., Crump et al., 2013; Germine et al., 2012).
2. *Do participants look at the distractor?* Previous research with salient distractors has found that they can produce oculomotor capture (e.g., Theeuwes et al., 1998). However, in other studies, participants actively avoid looking at salient distractors (e.g., Gaspelin et al., 2017; Moher et al., 2011; Zhang & Gaspelin, 2024; Zhang et al., 2025). Given the RT data in the target-present trials in Moher (2020) is broadly consistent with attentional capture, we predict that salient distractors will produce oculomotor capture.
3. *What types of errors do participants make?* Eye-movement data allow us to distinguish among two types of errors. There are search errors in which the participant fails to ever fixate the target, and decision errors in which the participant does fixate the target but fails to report its presence (e.g., Kundel et al., 1978). We predict that both types of errors will be present in this task and impacted by whether the distractor is fixated (see below).
4. *Do participants look at less of the image when a distractor is present?* One of the interpretations put forth by Moher (2020) is that when salient distractors are present, participants spend less time gathering information from the display. Therefore, we predict that when distractors are present, participants will fixate fewer objects and reduce the total area of the scene viewed by the participant.
5. *Does whether the participant looks at the distractor impact behavior?* Attentional capture is generally not a uniform process that occurs on every trial (e.g., Anderson & Folk, 2010). Therefore, there will be some trials where the eye fixates the distractor, and others where it does not. We predict that distractor-induced quitting patterns of key-press data will only be observed on the subset of trials in which the participant fixates the distractor.

## Methods

The experiment was pre-registered at https://osf.io/ft2z4/.^1^ Data from this study are available at that link. Analyses not described in the preregistration are labeled as exploratory.

### Participants

Forty-three participants (33 male, seven female, two non-binary, one unreported, mean age: 19.4 years) completed the study. Three participants were excluded from analyses for overall accuracy below 60% or accuracy below 10% in one of the four analysis bins comprising the possible combinations of target and distractor presence, using the same criteria as Moher (2020) and consistent with the preregistration plan. Moher (2020) found robust evidence of distractor-induced quitting with 75 participants. Since the current data were collected in the lab and the experiment was longer in duration, we aimed to collect 40 total participants. Based on the effect size of the RT interaction between target presence and distractor presence in Moher (2020), this gave us over 99% power to detect that same interaction in the current dataset according to calculations in G*Power (version 3.1). Participants were required to have normal or corrected-to-normal vision as well as normal color vision. The protocol was approved by the Connecticut College Institutional Review Board.

### Apparatus

Stimuli were displayed on an ASUS computer monitor using custom scripts written in MATLAB and Psychophysics Toolbox (Brainard, 1997). An Eyelink 1000 eye tracker was used to monitor the position of the left eye. Participants were seated approximately 92 cm away from the display, using a desk-mounted chinrest to stabilize head position. Key-press responses were recorded from a MilliKey SH-4 response box.

### Stimuli

**Fig. 1:**
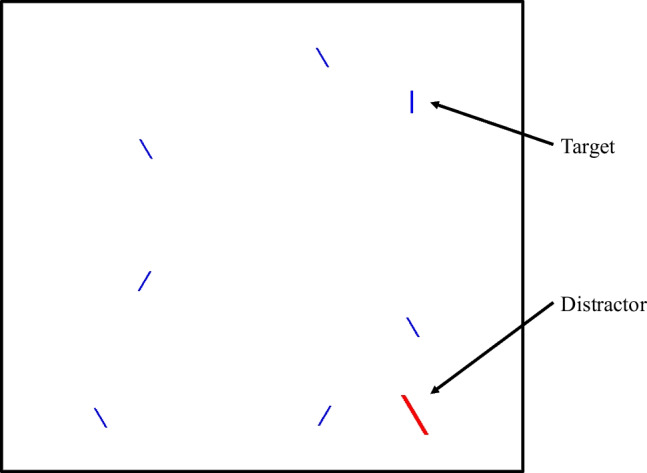
A sample trial on which a salient distractor was present, and a target (vertical line) was also present. The background in the experiment was black, but is changed to white here for visibility. “Target” and “Distractor” labels and arrows are for illustration purposes only and were not visible during the experiment

Stimuli were closely adapted from Moher (2020). On each trial, eight lines were presented on a black background (Fig. 1). These were primarily blue lines subtending approximately .09˚ of visual angle in width and .86˚ in height, each randomly tilted 30˚ to the left or right. On a randomly selected 50% of all trials, one of the lines was a target in the form of a vertical blue line of the same size. On an independently randomly selected 50% of all trials, one of the lines was a salient distractor. The salient distractor was four times as large, colored red,^2^ and appeared at a delayed onset of 100 ms. Any use of the term “distractor” in this study refers to a salient distractor unless otherwise noted. All other stimuli present in the visual search task that were not the target or salient distractor are referred to as non-target items unless otherwise noted.

Each line was placed at a randomly selected unique location in a 9 × 9 grid of possible locations, with the exception that no lines could appear along the central axis of either the horizontal or vertical dimension. Locations in the grid were equally spaced apart, and the grid subtended approximately 13.76˚ of visual angle horizontally and vertically. After each line was placed, it was randomly jittered up to 5 pixels in either direction along both the horizontal and vertical axes.

### Procedure

On each trial, a fixation cross was presented at the center of the display subtending approximately 0.52˚ of visual angle. After the participant stayed fixated within 2.58˚ of the center of the fixation cross for 0.5 s, the search stimuli appeared. The search display stayed visible until the participant responded with a keypress to indicate whether a target was present or absent. There was a 1-s intertrial interval during which a blank black screen was displayed.

Participants started the experiment with a 9-point eye-position calibration procedure. Following this, there were 12 practice trials with feedback, followed by three blocks of 100 trials each. After each block, 9-point calibration was repeated. In addition, prior to each trial, participants completed a drift correction to ensure accurate recording of eye position. Participants were not explicitly required to make eye movements. However, the stimuli were small and eye movements were likely required in most cases to locate the target; as evidence of this, participants made at least one eye movement on 96.7% of all trials.

### Data analysis

Eye movements were defined by predetermined velocity and acceleration thresholds in the Eyelink 1000 manufacturer settings, with a saccade defined as a sample with a velocity exceeding 30.0°/s and acceleration exceeding 8,000°/s. An object was considered fixated if the eye fell within a radius of approximately 1.5° of the center of the object.^3^

All trials with RTs longer than 10 s or shorter than 200 ms were eliminated from analyses, and fixations. For RT data, only correct trials are included in reported analyses. RT outliers were trimmed using a recursive trimming procedure based on standard deviations (Van Selst & Jolicœur, 1994). Fixations with durations of less than 60 ms were removed from eye-movement analyses.

For error types, we distinguished between *search errors*, in which the target was fixated, and *decision errors*, in which the target was fixated but not reported (e.g., Kundel et al., 1978). *Search coverage* was defined as the approximate number of pixels viewed in a given display, and was used as an estimate of the total area of the display viewed on any given trial. This value was calculated by creating a grid of all pixels on the display, and for each fixation, filling in all pixels within a diameter of 2.1° of visual angle of that fixation (e.g., Drew et al., 2012).

## Results and discussion

For the following analyses, unless otherwise noted, we conducted 2 × 2 ANOVAs with factors of target (present vs. absent) and distractor (present vs. absent). For significant interactions, we conducted simple main-effects analyses to parse the interactions. Analyses were performed using JASP software (JASP Team, 2025).

**Fig. 2:**
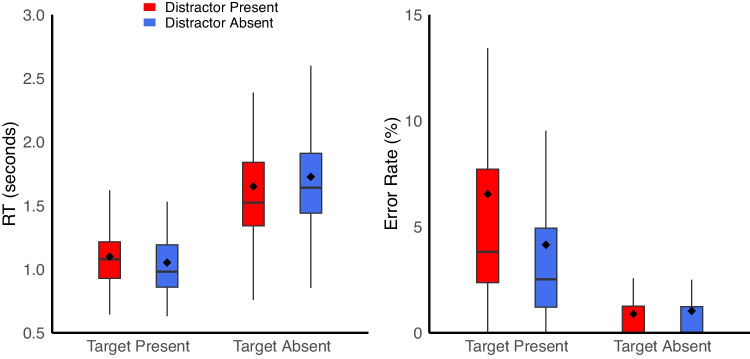
Behavioral data replicating Moher (2020). **Left:** On target-present trials, response times (RTs) were longer when distractors were present than when they were absent. On target-absent trials, RTs were longer when distractors were absent. **Right:** On target-present trials, miss errors were more frequent when distractors were present. Solid lines reflect the median, dots reflect the mean, and the edges of the box reflect the first and third quartile of the data. Charts generated with the geom_boxplot function in RStudio (Posit Team, 2023)

*Does distractor-induced quitting replicate in a lab-based setting?* First, we examined key-press data using the same approach as Moher (2020). RTs were longer on target-absent trials (1,688 ms) compared to target-present trials (1,076 ms), *F*(1,39) = 93.13, *p* <.001, η_p_^2^ =.71. There was no main effect of distractor, *F*(1,39) = 1.32, *p* <.26, η_p_^2^ =.03. However, there was an interaction between target and distractor, *F*(1,39) = 24.29, *p* <.001, η_p_^2^ =.38. On target-present trials, RTs were longer when distractors were present (1,099 ms) compared with when they were absent (1,052 ms), *F*(1,39) = 10.36, *p* =.003. Conversely, on target-absent trials, RTs were shorter when distractors were present (1,650 ms) compared with when they were absent (1,726 ms), *F*(1,39) = 13.79, *p* <.001. In other words, on target-present trials the usual pattern of attentional capture was observed, but on target-absent trials participants responded more quickly when distractors were present (Fig. 2, left).

We also compared latency to fixate the target on this subset of trials, and found that target-fixation latency was longer when salient distractors were present (555 ms) compared to when they were absent (518 ms), t(39) = 2.99, p =.005, Cohen’s *d* =.47. This suggests that the effect observed in the RT data is not about decision time alone, but instead that the presence of a salient distractor delays the time it takes the participant to find the target.

For accuracy data, there were higher error rates on target-present trials (5.3%) compared to target-absent trials (1.0%), *F*(1,39) = 20.38, *p* <.001, η_p_^2^ =.34. There were also higher error rates when salient distractors were present (3.7%) compared to when they were absent (2.6%), *F*(1,39) = 13.25, *p* <.001, η_p_^2^ =.25. Finally, there was a significant interaction, *F*(1,39) = 12.86, *p* <.001, η_p_^2^ =.25. On target-absent trials, there was no difference between distractor-present (0.9%) and distractor-absent (1.0%) error rates, *F*(1,39) = 0.33, *p* =.57, reflecting the rarity of false alarms within the task. However, on target-present trials, errors were significantly higher when salient distractors were present (6.5%) compared to when they were absent (4.2%), *F*(1,39) = 15.131, *p* <.001 (Fig. 2, right).

Taken together, these data broadly replicate the results of Moher (2020) and others (e.g., Lawrence & Pratt, 2022). This pattern of data is consistent with the idea that when salient distractors are present, participants quit search earlier than they otherwise would – distractor-induced quitting. As a result, when distractors are present, participants have shorter RTs on target-absent trials but higher miss rates on target-present trials. Next, we examine eye movements to gain a better understanding of the mechanism of this early quitting effect.

**Fig. 3:**
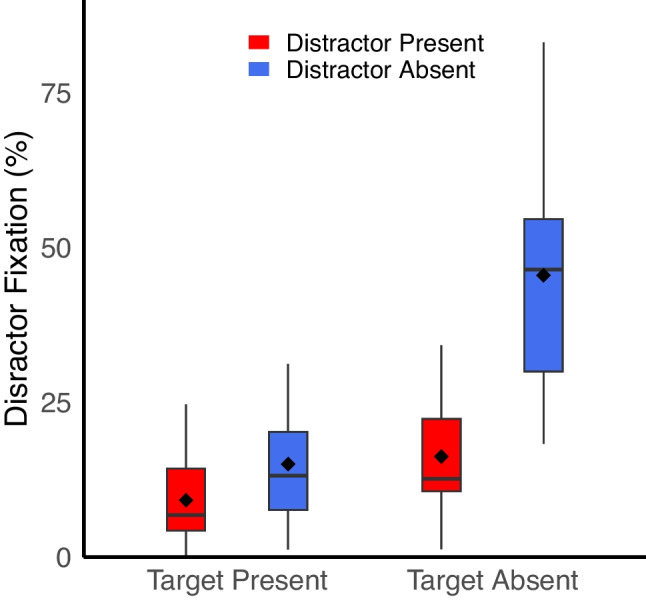
On distractor-absent trials, distractor fixation refers to the probability of fixating the “dummy distractor,” which is an arbitrarily selected non-target. On both target-present and target-absent trials, participants were more likely to fixate the dummy distractor on a distractor-absent trial than the salient distractor on a distractor-present trial, indicating that participants were actively avoiding fixating the salient distractor. Solid lines reflect the median, dots reflect the mean, and the edges of the box reflect the first and third quartile of the data. Charts generated with the geom_boxplot function in RStudio (Posit Team, 2023)

*Do participants look at the distractor?* To assess oculomotor capture, we used two measures. First, for distractor-absent trials, we randomly selected one non-target item to serve as a “dummy” distractor. The “dummy” distractor was selected independently on each trial and could be at any of the locations on the display. This allowed us to compare how frequently participants fixated the distractor when it was actually a salient distractor compared to when it was just another non-target. There was a main effect of target presence, with a higher probability of fixating the distractor (either the actual salient distractor on distractor-present trials, or the dummy distractor on distractor-absent trials) when the target was absent (30.9%) compared to when it was present (12.1%), *F*(1,39) = 255.72, *p* <.001, η_p_^2^ =.87. More critically, there was a main effect of distractor, with fixation being more likely on the dummy distractor on distractor-absent trials (30.3%) compared to the actual salient distractor on distractor-present trials (12.7%), *F*(1,39) = 91.34, *p* <.001, η_p_^2^ =.70. Finally, there was a significant interaction, *F*(1,39) = 151.53, *p* <.001, η_p_^2^ =.80. On target-present trials, there was a relatively modest difference in fixation rates for the dummy distractor on distractor-absent trials (15.0%) compared to salient distractor on distractor-present trials (9.2%). On target-absent trials, there was a larger gap, with a 45.5% fixation rate on distractor-absent trials compared to a 16.2% fixation rate for distractor-present trials. In both cases, the difference between distractor-present and distractor-absent trials was significant, *p*s <.001 (Fig. 3).

These data clearly demonstrate that not only did the salient distractor fail to capture the eyes on a regular basis, but instead participants actively avoided fixating the salient distractor, contrary to our predictions. As a second measure, for all distractor-present trials, we calculated the frequency that a participant fixated the salient distractor in each target condition and divided that number by the average frequency that the participant fixated each of the remaining non-target, non-salient distractor items on those trials. This latter term represents the probability that a participant would fixate a typical non-target, non-salient distractor on a given trial. A number above 1 for this calculated ratio indicates preference for fixating the salient distractor relative to other non-targets, while a number below 1 indicates a preference for fixating the non-salient non-targets.

A paired-samples t-test revealed that this ratio was higher on target-present trials (.69) compared to target-absent trials (.41), *t*(39) = 2.83, *p* =.007, Cohen’s *d* =.45. In other words, there was a stronger preference for fixating non-salient non-targets on target-absent compared to target-present trials. Notably, both numbers were significantly lower than 1, *p*s <.05, indicating that regardless of target presence, participants were avoiding fixating the salient distractor relative to other search objects. Taken together, both of these analyses demonstrate that not only did we not observe oculomotor capture, we observed suppression of eye movements directed towards the salient distractor (e.g., Gaspelin et al., 2017; see General discussion for more detail).

**Fig. 4:**
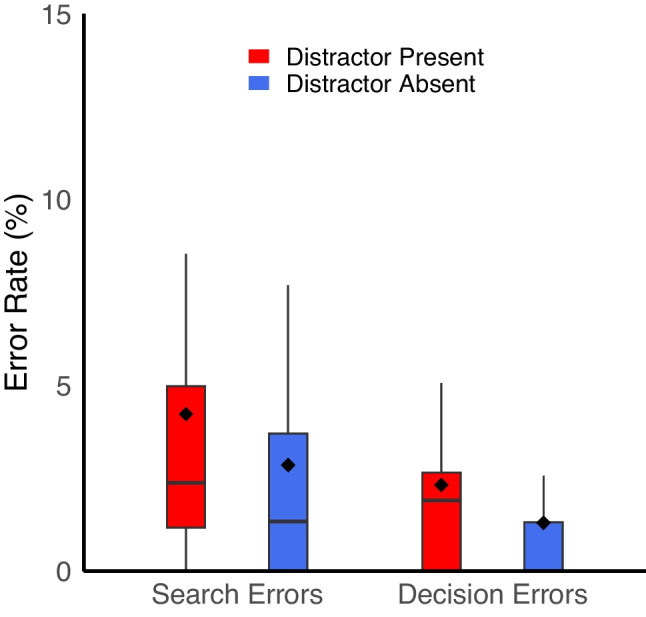
The presence of a salient distractor increased both search error and decision error frequency. Solid lines reflect the median, dots reflect the mean, and the edges of the box reflect the first and third quartile of the data. Charts generated with the geom_boxplot function in RStudio (Posit Team, 2023)

*What types of errors do participants make?* To better understand the types of errors that occur when targets are present, we conducted a 2 × 2 ANOVA with factors of error type (search error vs. decision error) and distractor presence (present vs. absent) for target-present trials only. Search errors were about twice as frequent (3.5%) as decision errors (1.8%), *F*(1,39) = 12.53, *p* =.001, η_p_^2^ =.24. Consistent with earlier analyses, errors were also more frequent on distractor-present trials (3.3%^4^) compared to distractor-absent trials (2.1%), *F*(1,39) = 15.13, *p* <.001, η_p_^2^ =.28. There was no significant interaction, *F*(1,39) = 0.23, *p* =.64, η_p_^2^ =.01 (Fig. 4).

These data suggest that participants commit both types of errors, but that search errors in which participants fail to ever fixate the target before terminating the trial are more common than decision errors in which they fixate the target but fail to report its presence. Furthermore, the presence of a distractor increases overall error frequency, but does not appear to change which type of error occurs more or less often. Another way to consider this is that distractors increase the frequency of both types of errors; we consider the implications of this finding in greater detail in the *General discussion*.

*Do participants look at less of the image when a distractor is present?* One interpretation of the keypress data in Moher (2020) is that when a salient distractor is present, participants gather less information from the display, and that is why they are more likely to miss targets. To test this possibility, we examined the total number of fixations on objects that were not the target across all conditions. There were fewer average total fixations when targets were present (1.18) compared to when they were absent (3.48), *F*(1,39) = 144.75, *p* <.001, η_p_^2^ =.79, as expected given significantly shorter RTs on target-present compared to target-absent trials. There was also a main effect of distractor presence on fixations, *F*(1,39) = 8.02, *p* =.007, η_p_^2^ =.17, best explained by a significant interaction, *F*(1,39) = 32.95, *p* <.001, η_p_^2^ =.46. When targets were present, there was no significant difference in total fixations between distractor-present (1.21) and distractor-absent (1.15) trials, *F*(1,39) = 1.45, *p* =.24. However, when targets were absent, fewer fixations occurred when distractors were present (3.31) than when they were absent (3.59), *F*(1,39) = 30.85, *p* <.001.

These data indicate that on target-absent trials, participants indeed searched less of the display when distractors were present. To complement this analysis, we examined screen coverage as a function of target and distractor presence. More pixels were viewed when targets were absent (68,577) compared to when they were present (35,745), *F*(1,39) = 166.53, *p* <.001, η_p_^2^ =.81. For distractors, more pixels were viewed when distractors were absent (52,938) compared to when they were present (51,384), *F*(1,39) = 8.18, *p* =.007, η_p_^2^ =.17. There was also a significant interaction, *F*(1,39) = 51.89, *p* <.001, η_p_^2^ =.57. On target-present trials, more pixels were viewed when a distractor was present (36,493) compared to when a distractor was absent (34,996), *F*(1,39) = 5.51, *p* =.02. However, on target-absent trials, the opposite pattern was observed – more pixels were viewed when a distractor was absent (70,880) compared to when a distractor was present (66,275), *F*(1,39) = 39.14, *p* <.001.

This outcome supports the notion that when distractors were present, participants viewed less of the display on target-absent trials. However, one possible explanation for the difference is simply that participants were avoiding fixating the salient distractor, and thus while they searched less of the display, it should not have impacted their ability to find the target since they were only avoiding a known non-target object (the salient distractor). In a separate exploratory analysis where we compared the total number of fixations on non-target, non-distractor objects (eliminating fixations on the dummy distractor for distractor-absent trials), there was indeed no interaction between target and distractor for total fixations, *F*(1,39) = 1.28, *p* =.27, η_p_^2^ =.03.

If participants were simply avoiding the distractor, why did error rates increase? One possible explanation is that fixations on non-objects differed as a function of distractor presence, since our prior analysis focused only on fixations that fell within the areas of interest of search objects. To examine this possibility, in an exploratory analysis, we analyzed the proportion of non-object fixations as a function of target and distractor presence. There was no main effect of distractor, *F*(1,39) = 3.85, *p* =.06, η_p_^2^ =.09. There were more non-object fixations on target-absent trials (2.37) compared to target-present trials (1.09), *F*(1,39) = 127.59, *p* <.001, η_p_^2^ =.77. Critically, there was a significant interaction, *F*(1,39) = 12.44, *p* =.001, η_p_^2^ =.24. On target-present trials, there was no significant difference between distractor-present (1.11) and distractor-absent (1.06) non-target fixations, *F*(1,39) = 3.14, *p* =.08. However, on target-absent trials, non-target fixations were less frequent when distractors were present (2.28) compared to when they were absent (2.45), *F*(1,39) = 9.67, *p* =.003. Notably, while non-object fixations were not quite as frequent as object fixations, they did make up a substantial portion of behavior, suggesting that participants were saccading to locations between objects with some frequency.

**Fig. 5:**
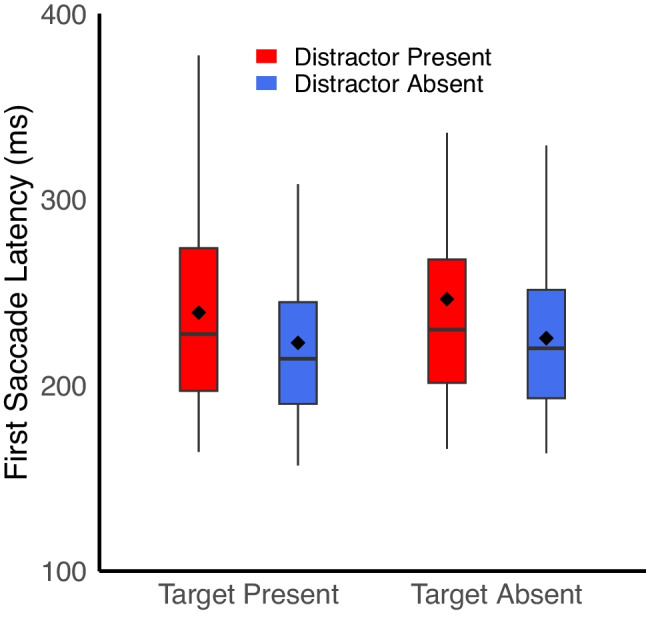
Time to initiate the first saccade was longer for distractor-present trials compared to distractor-absent trials. Solid lines reflect the median, dots reflect the mean, and the edges of the box reflect the first and third quartile of the data. Charts generated with the geom_boxplot function in RStudio (Posit Team, 2023)

Thus, even taking into account the active avoidance of the salient distractor, participants made fewer fixations between objects when distractors were present. We also examined first saccade latencies to better understand if distractors were impacting the onset of search. First saccade latencies were longer when distractors were present (243 ms) compared to when they were absent (224 ms), *F*(1,39) = 33.05, *p* <.001, η_p_^2^ =.46. There was no main effect of target presence on first saccade latencies, *F*(1,39) = 3.92, *p* =.055, η_p_^2^ =.09, nor an interaction, *F*(1,39) = 1.55, *p* =.22, η_p_^2^ =.04 (Fig. 5). Other studies have shown that rapidly initiated saccades are more likely to go to salient distractors when they are present (e.g., van Zoest & Donk, 2005). Thus, in the current study, participants may have been waiting out the short-lived salience wave (e.g., Donk & van Zoest, 2008) in order to avoid fixating the distractor when it was present. Alternatively, they may have first briefly attended the distractor before inhibiting it, preventing a saccade but taking additional time before searching the remainder of the display (e.g., Geng, 2014; Moher & Egeth, 2012).

One possible interpretation of the fixation and initial saccade latency data is that when distractors are present, participants actively inhibit the distractor, and this process requires working memory resources (e.g., Lavie & de Fockert, 2005). This increase in working memory load may delay search initiation and disrupt search guidance. These ideas are discussed at greater length in the *General discussion*.

*Does whether the participant looks at the distractor impact behavior?* Finally, we were interested in the extent to which behavior differs depending on whether the participant fixated the distractor on a given trial. To examine this, we conducted a 2 × 2 ANOVA with factors of distractor fixation (fixated vs. not fixated) and target presence on both RT and error rate, using only distractor-present trials. One participant was excluded from analysis because they did not have trials in which they fixated the distractor in one of the conditions.

As expected, RTs were longer when targets were absent (1,780 ms) than when they were present (1,232 ms), *F*(1,38) = 58.37, *p* <.001, η_p_^2^ =.61. RTs were also longer when the distractor was fixated (1,678 ms) than when it was not (1,334 ms), *F*(1,38) = 91.19, *p* <.001, η_p_^2^ =.71. However, there was no interaction, *F*(1,38) = 0.27, *p* =.61, η_p_^2^ =.01. In other words, fixating the distractor led to longer overall RTs, regardless of condition, suggesting that early quitting is not a consequence of fixating the distractor itself as we predicted. Instead, on the subset of trials where the distractor is fixated, RTs are longer regardless of target presence. However, as we know from our analyses reported above, the subset of trials on which the distractor is fixated is relatively small.

Errors were more frequent when targets were present (9.4%) compared to when they were absent (0.9%), *F*(1,38) = 27.86, *p* <.001, η_p_^2^ =.42. Errors were also more frequent when the distractor was fixated (7.0%) compared to when it was not (3.4%), *F*(1,38) = 7.64, *p* =.009, η_p_^2^ =.17. There was also an interaction, *F*(1,38) = 7.38, *p* =.01, η_p_^2^ =.16. On target-present trials, there were more errors when the distractor was fixated (13%) compared to when it was not (5.8%), *F*(1,38) = 7.76, *p* =.008. However on target-absent trials, there was no difference in error rates as a function of whether the distractor was fixated (1.0%) or not (0.9%), *F*(1,38) = 0.02, *p* =.89. An analysis examining the types of errors impacted by whether or not the distractor was fixated showed no main effect of error type, nor an interaction between error type and distractor presence, *p*s >.52.^5^

These data show that fixating the distractor caused a significant increase in miss errors. Does this explain the entire effect of distractor presence on miss errors? As an exploratory analysis, we directly compared the rate of miss errors on distractor-present trials where no fixation on the distractor occurred against the rate of miss errors on all target-present trials in which a distractor was absent. We found there were still significantly more errors on distractor-present (5.8%) compared to distractor-absent (4.2%) trials, *t*(39) = 2.89, *p* =.006, Cohen’s *d* =.46. In other words, even when the distractor is not fixated, the presence of that distractor increases misses.

### Exploratory analysis

One consideration regarding the avoidance of the distractor is whether this is behavior that emerges immediately, or takes time to develop. For example, there is evidence to suggest that salience may influence attention more during the initial parts of search, or that distractor suppression can function in a reactive rather than proactive manner in some contexts (e.g., Addleman & Störmer, 2022; Donk & van Zoest, 2008; Geng, 2014; Moher & Egeth, 2012). Thus, it is possible that distractor avoidance has different characteristics over the course of a trial.^6^

To examine this possibility, we analyzed the probability that the distractor was fixated on each of the first four fixations for each trial type. For trials with fewer than four total fixations, data were included up to the final fixation for that trial. One participant was excluded from this analysis for having no observations in one of the conditions. We conducted a 4 × 2 × 2 ANOVA with factors of fixation number (1–4), target presence, and distractor presence and distractor fixation as the dependent variable. As in earlier analyses, one non-target item was arbitrarily assigned as the dummy “distractor” on each distractor-absent trial.

There were main effects of target and distractor, *p*s <.001, consistent with prior analyses. There was also a main effect of fixation number, *F*(3,114) = 21.78, *p* <.001, η_p_^2^ =.36, with a lower probability of fixating the distractor on later fixations compared to earlier fixations. Critically, there was an interaction between fixation number and distractor, *F*(3,114) = 20.83, *p* <.001, η_p_^2^ =.35.

To parse this interaction, we conducted simple main effects analyses on the impact of distractor presence for each fixation number. There was a significant effect of distractor at fixations 2–4, *p*s <.001, with fewer fixations on the salient distractor on distractor-present trials compared to fixations on the dummy “distractor” on distractor-absent trials in each case. In other words, for fixations 2–4, participants were actively avoiding the salient distractor. However, on the first fixation, there was no effect of distractor presence, *F*(1,38) = 0.058, *p* =.81. This suggests there was no distractor avoidance (but also no oculomotor capture) on the first fixation; the salient distractor was indistinguishable from other non-targets in terms of how it impacted the eyes for this fixation.

There was also an interaction between target and distractor, along with a three-way interaction, *p*s <.05. These interactions likely reflect the impact of target saccades, in which a proportion of overall saccades when a target is present are more likely to be directed to the target compared to any other item.

**Fig. 6:**
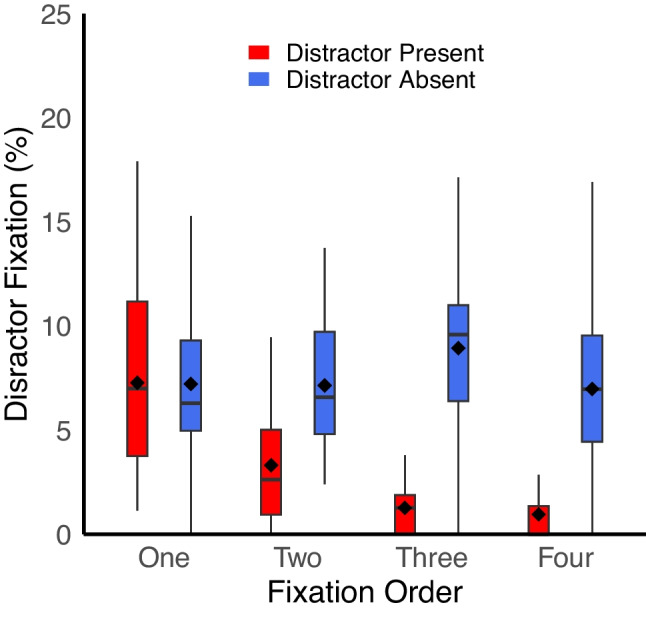
Salient distractors were no more or less likely to capture the eyes for the first saccade of each trial, but on subsequent saccades participants were actively avoiding fixating the salient distractor (on distractor-present trials) compared to the dummy distractor (on distractor-absent trials). Solid lines reflect the median, dots reflect the mean, and the edges of the box reflect the first and third quartile of the data. Charts generated with the geom_boxplot function in RStudio (Posit Team, 2023)

This is a relatively complex analysis but the impact is most clearly observed when comparing saccades to the salient distractor against saccades to the “dummy” distractor on target-absent trials, as these trials are not impacted by saccades to a target since no target is present. As can be seen in Fig. 6, it is clear that suppression of the distractor emerges not immediately, but only after the initial saccade. One potential implication of these results is that the disruption of search performance observed in earlier analyses is a consequence of the continued effort to suppress the distractor throughout the trial.

## General discussion

Consistent with earlier findings (Moher, 2020), when salient distractors were present, RTs were shorter on target-absent trials and error rates were higher on target-present trials. This lab-based replication is consistent with distractor-induced quitting. As predicted, we also found that there were fewer total fixations and reduced screen coverage when distractors were present. However, a key driver of this effect was unexpected – participants actively avoided fixating the salient distractor when it was present.

Does this mean that participants were effectively suppressing the distractor? Previous work has shown that the frequency of eye movements to salient distractors can be reduced based on factors such as distractor probability (e.g., Moher et al., 2011; Sayim et al, 2010). Furthermore, in some contexts, oculomotor suppression is observed, in which fewer eye movements are directed towards salient distractors compared even to other non-salient, non-targets (e.g., Gaspelin et al., 2017; Zhang & Gaspelin, 2024; Zhang et al., 2025).

The present study demonstrated a similar oculomotor suppression effect of a salient distractor. Notably, however, suppression in the current study was not immediate, proactive suppression as in Gaspelin et al. (2017), but instead emerged reactively starting with the second fixation according to our exploratory analysis. Furthermore, the presence of the distractor also came with clear, measurable costs in behavior. First, miss errors were more frequent when distractors were present, for both search and decision errors. Second, fewer non-object fixations occurred when distractors were present. Third, initial saccade latencies were delayed when distractors were present. Fourth, RTs were longer when distractors were present on target-present trials. Altogether, these data demonstrate that the way in which salient distractors disrupt search in this particular type of task is not through oculomotor capture, but instead through a combination of behavioral changes.

How can we better understand this series of changes elicited by a salient distractor? One possible explanation is that the distractor produces an increase in working memory load. There is a possibility that attempting to suppress the distractor depletes working memory or other cognitive resources, resulting in disrupted search guidance. This is consistent with our exploratory analysis demonstrating that the distractor is actively avoided throughout the trial after the initial saccade.

Similar to distractor avoidance (e.g., Lavie & de Fockert, 2005), search guidance requires working memory resources and guidance can be disrupted when working memory is overloaded (e.g., Berggren & Eimer, 2018; Zhang et al., 2011) or by the contents of working memory (e.g., Beck & Vickery, 2019; Downing, 2000, though see Drew et al., 2016, for a search task where working memory load does not appear to interfere). Furthermore, search templates can be impacted by the contents of working memory, guiding attention towards or away from specific features or locations (see, e.g., Carlisle, 2023, for a review). A disruption in guidance could explain the reduction of saccades that fall in-between items, as these saccades might plausibly aid in the guidance of the next saccade by helping the observer take in information about multiple potential search targets. This is consistent with prior work that has suggested that more complex search tasks with more potential targets lead to a smaller “useful field of view” and shorter saccades (Drew et al., 2017). Furthermore, RT data showed that distractors increased search times when targets were present, even though fixations to the distractor itself were being avoided. Some of that may be explained by delayed initial saccades, but the magnitude of that difference on target-present trials (16 ms) was much smaller than the overall distractor interference effect in the RT data on target-present trials (47 ms), again consistent with less effective search guidance. Finally, the increase in search errors when distractors were present suggests that guidance was impacted by the presence of a salient distractor. The increase in decision errors, though not related to guidance, would also be consistent with an increased working memory load disrupting object processing (Woodman & Luck, 2004).

According to this interpretation, the presence of a salient distractor decreased guidance in search. On target-present trials, this led to a delay in finding the target. On target-absent trials, this led to participants terminating the trial earlier than they did when distractors were absent. This interpretation is broadly consistent with the idea that distractors produce early quitting in visual search (Moher, 2020). Participants do indeed quit earlier when distractors are present, and they do miss more targets as a result. The eye tracking in the present study has uncovered potential specific mechanisms that lead to this early quitting.

In future research, a direct examination of working memory and search guidance could shed further light on how distractors disrupt search. For example, increasing working memory load through other means may produce similar impacts on eye movements and quitting behavior as the presence of a salient distractor. The specific features of the distractor may also impact guidance, dependent upon how much those features interact with templates in working memory. The salient distractors in the present study were abrupt onsets. Previous work has shown that abrupt onsets are particularly potent in capturing attention (e.g., Jonides & Yantis, 1988) and do not lead to proactive oculomotor suppression, unlike color singletons (e.g., Adams et al., 2023). However, Lawrence et al. (2023a) demonstrated that early quitting effects were observed even when distractors were not presented at a delayed onset in a search task that was otherwise similar to the current experiment. Thus, future eye-tracking studies that vary whether the distractor is presented at a delayed onset may be important in order to better understand the role of distractor features in early quitting. Another possible avenue for future research is to examine other factors that might trigger these changes in search strategy. For example, increased physical effort has been shown to speed search but produce greater distractor interference (e.g., Park et al., 2021), and thus might also produce a similar pattern of eye-movement behavior in the type of search task used in the present study.

Finally, future research may involve targeted approaches to understanding the way in which capture, suppression, and guidance change throughout the course of a trial. Our exploratory analysis here suggested that avoidance of the distractor is not present initially, but comes online after the first saccade is executed. Is there a shift of attention to the distractor that precedes suppression (e.g., Moher & Egeth, 2012), or is there a subset of trials on which no shift of attention occurs, but the signal from the distractor allows for proactive suppression of the distractor (e.g., Sawaki & Luck, 2010)? The latter explanation might suggest a direct relationship between the strength of the signal from the distractor, and the subsequent impact on early quitting.^7^ Complementary methods such as electroencephalography and visually guided reach-tracking may help understand the role that selective attention plays during the early parts of this type of search.

How might these findings impact real-world problems? There are numerous examples of visual search tasks in which targets are not always present, such as x-ray baggage security or medical image screening (e.g., Biggs et al., 2018). Frequently in these types of searches, there will be a subset of images that contain a salient item that is not the target. It is quite plausible that the effects we observed here also impact these types of real-world searches. In that case, it would be useful to better understand the impacts of these salient distractors on search and identify strategies for observers in these scenarios to avoid early quitting. In the present study the salient object is always a non-target, suggesting that even when they are completely irrelevant, salient distractors can disrupt search and produce early quitting. With increasing use of artificial intelligence, it is likely that there are also many domains in which salient cues will be used in difficult searches to indicate potential targets. For example, computer-aided detection has already been in place in medical diagnostic imaging for many years (e.g., Doi, 2007), but there is great controversy around the efficacy of cueing in this manner in clinical practice (e.g., Fenton et al., 2011; Kohli & Jha, 2018). Recent work from our lab demonstrates that salient cues can also produce early quitting on the subset of trials in which those cues fail to highlight the target (Moher et al., 2024). Thus, salient signals in general appear capable of triggering early quitting in a variety of contexts, suggesting that a careful consideration of their role in real-world search is important to avoid critical search errors.

In summary, participants actively avoided fixating a salient distractor in a simple visual search task. However, the presence of this distractor produced clear behavioral costs. Saccade initiation was delayed, target RT was delayed, fewer fixations between items occurred, and there was an increase in both search errors and decision errors. These results underscore how distractions can disrupt search even when they fail to capture the eyes.

## Data Availability

Methods for both experiments were preregistered at https://osf.io/ft2z4/ and all subject-level data are available at that site.

## Footnotes

1 We initially pre-registered a version of the task in which distractor onset was not delayed, but partway through data collection observed that the distractor was producing minimal effects on behavior. Due to relatively limited resources, we decided to terminate that experiment and instead focus on a more direct replication of Moher (2020).

2 The RGB color values in Matlab were blue: [0, 0, 255] and red: [255, 0, 0].

3 We adjusted this from the 2° proposed in the preregistration after viewing areas of interest (AOis) overlaid on stimulus images, because this prevented AOIs from overlapping. This adjustment was made prior to examining any fixation data.

4 Note that these are marginal means being reported. To infer the total error rate combining the two types of errors, you would multiply these numbers by two.

5 Note that earlier analyses showed that search errors were more frequent than decision errors. Here, numerically, that was true when the distractor was not fixated (search errors: 3.9%, decision errors: 2.0%), whereas both types were equally frequent when the distractor was fixated (6.5%). Because distractor fixations were relatively rare overall, and the subset of trials on which a distractor was fixated and an error was made is even smaller, the data for this analysis are likely too noisy to detect whether there is a meaningful difference here. Future research targeted towards this specific question might better be able to determine whether fixating the distractor produces changes in specific types of errors, but in our current analyses we can draw no clear conclusions.

6 We thank Nick Gaspelin for raising this possibility.

7 We thank Nick Gaspelin for this suggestion.

## Code availability

We prefer to offer analysis code upon request rather than post on the Open Science Framework.
